# A 12 month review of a modified protocol using low dose Dabigatran Etexilate in postoperative thromboembolic prophylaxis in joint replacement surgery

**DOI:** 10.1186/1477-9560-10-14

**Published:** 2012-08-17

**Authors:** Padmanabhan Subramanian, Shanjitha Kantharuban, Sophie Shilston, Oliver James Pearce

**Affiliations:** 1 North East Thames London orthopaedic rotation, Current hospital: Whipps Cross Hospital, Whipps Cross Road, London E11 1NR, UK; 2 Core Surgical Trainee Year 1, Milton Keynes Hospital, Eaglestone, Milton Keynes, MK6 5LD, UK; 3 Consultant Trauma & Orthopaedic Surgeon, Milton Keynes Hospital, Eaglestone, Milton Keynes, MK6 5LD, UK

**Keywords:** Dabigatran, VTE prophylaxis, Arthroplasty, DVT, PE

## Abstract

**Background:**

Venous Thrombo-embolic disease is currently a hot topic especially in the UK. 25,000 patients per year die of Pulmonary Emboli (PE) in the United Kingdom (UK). Hip and knee arthroplasty surgery is associated with an increased rate of deep vein thrombosis (DVT) and pulmonary embolus (PE). The National Institute for Clinical Excellence (NICE) guidelines introduced in January 2010 recommended use of subcutaneous heparin or an oral anticoagulant (Dabigatran or Rivaroxiban) for 10-14 days post knee and 28-35 days post hip arthroplasty. In our unit we were keen on the advantages of an oral anticoagulant post arthroplasty in terms of patient compliance, and avoiding the need for self administered injection in the community.

**Methods:**

We analysed all the notes, blood results and imaging of patients undergoing total hip or knee arthroplasty and present 1 year’s data using a regime of subcutaneous Dalteparin whilst an inpatient, followed by discharge on oral Dabigatran at a low dose (150 mg once daily).

**Results:**

There were 337 patients over 1 year with hip and knee arthroplasty, with a 1.19% rate of DVT with no PEs and 1 death due to an unrelated cause. There was a transfusion rate of 11.57% with 1.19% patients taken back to theatre for evacuation of haematomas. There were no reported adverse effects of Dabigatran.

**Conclusion:**

Our treatment protocol is a novel practical approach for VTE prophylaxis in hip and knee replacement patients. This approach shows promising data but no definitive evidence to warrant wide-spread use of this new regime. This data can act as a foundation for larger randomised clinical trials.

## Introduction

Venous thrombo-embolic (VTE) disease is currently an important hot topic, especially in the United Kingdom. In response to growing political pressures, including alarming statistics such as 25,000 hospital deaths in patients per year due to PEs in the UK [1], new NICE guidelines were introduced in January 2010 for VTE prophylaxis [2].

These guidelines recommended use of subcutaneous low molecular weight heparin or an oral anticoagulant (Dabigatran Etexilate or Rivaroxiban) for 10-14 days post knee arthroplasty and 28-35 days post hip arthroplasty.

It is recognised that hip and knee arthroplasty are associated with an increased rate of VTE [2,3]. Our department designed an anticoagulant prophylaxis regime in response to these guidelines based on the following factors.

We were keen on the advantages of an oral anticoagulant post hip or knee arthroplasty in terms of patient compliance [4,5], and avoiding the need for self administered injection in the community. Dabigatran Etexilate (hereafter termed Dabigatran) has been tested in large clinical trials and has been shown to have equivalent effectiveness to subcutaneous Enoxaparin, and to be similar in terms of safety and side effect profile [6-9].

Data from the dose selection studies for Dabigatran demonstrated that an increase in the risk of bleeding on the day of surgery was associated with an increase in Dabigatran peak concentration, hence supporting the licenced protocol of halving the dose of Dabigatran on the day of surgery [10]. Furthermore, anecdotally, there were concerns with reference to wound discharge when oral anticoagulants were used in the immediate post operative period, so the decision was made to continue with the subcutaneous anticoagulant we normally used (Dalteparin 5000 units once daily) during the inpatient stay, and to discharge the patient home on oral Dabigatran for the remainder of the recommended timeframes.

There are two dosing levels of Dabigatran: 220 mg is the standard dose, with a recommendation for patients over the age of 75 years and/or ‘moderate’ renal failure (creatinine clearance 30-50 ml/min), and/or patients on concomitant Amiodarone and Verapamil, to have the lower dose of 150 mg daily [11]. The decision was made to use the low dose (150 mg) for all patients, as a large proportion of our patients were either over the age of 75 years or had moderate renal impairment, and this would simplify prescribing regimes for the high turnover of departmental junior doctor staff. The aim of this study therefore was to evaluate the outcomes of our VTE protocol and compare the results to the published literature.

## Materials and methods

All patients undergoing primary hip or knee arthroplasty between March 2010 and March 2011 were prescribed the aforementioned regime post operatively (Dalteparin 5000 u subcutaneously whilst an inpatient, and Dabigatran 150 mg once daily orally on discharge for 14 days for knees and 28 days for hips). The Dalteparin commenced the evening of the day of surgery at 1800 for morning operations, and at 0800 the following morning for evening operations.

Data concerning clinical DVT rate, clinical PE rate, mortality, wound ooze/discharge, Haemoglobin (Hb) drop, transfusion rate, infection rate and other complications was collected prospectively, and reviewed by an independent observer at 1 year. The patient population was operated upon by 7 Consultants and 2 Associate Specialists in a standard District General Hospital (DGH) setting.

The types of implants (by individual surgeon preference) were, for knees: Cemented Scorpio knees (Stryker, Newbury, UK), Uncemented LCS knees (Depuy, Leeds, UK). For the hips: Cemented Exeter (Stryker), and Uncemented Trident-Accolade (Stryker) or Uncemented Furlong (JRI Ltd, Sheffield, UK). All knee replacements had a re-transfusion drain (Bellovac, Astra Tech Healthcare, Gloucestershire UK) and received autotransfusion if more than 200 ml collected in the drain during the first 6 hours post operatively.

The following patients were excluded from this study on the basis of bias or because these patients were contra-indicated to receiving Dabigatran for thrombo-prophylaxis: Patients on Warfarin preoperatively, Patients with bleeding diathesis, patients requiring revision hip or knee arthroplasty, patients with deranged liver function tests (Liver enzyme blood tests: Alanine transaminase, Alkaline phosphatase and Aspartate transaminase, which were greater than 2 units above the upper normal limit), patients on Quinidine and patients with renal failure with a creatinine clearance of less than 30 ml/min. There were a total of 58 patients (14.7%) excluded from this study (40 were due to revision arthroplasty).

To ensure all possible clinical thromboembolic events were recorded, the hospital databases for the VTE clinic (where all patients with DVT or PE are treated locally), PACS radiology system for positive duplex and computer tomography assisted pulmonary angiogram scans and the electronic medical notes for all patients were all reviewed by an independent observer (a qualified orthopaedic surgical trainee MBBS, MRCS). The data collection was complete.

## Results

**Figure 1:**
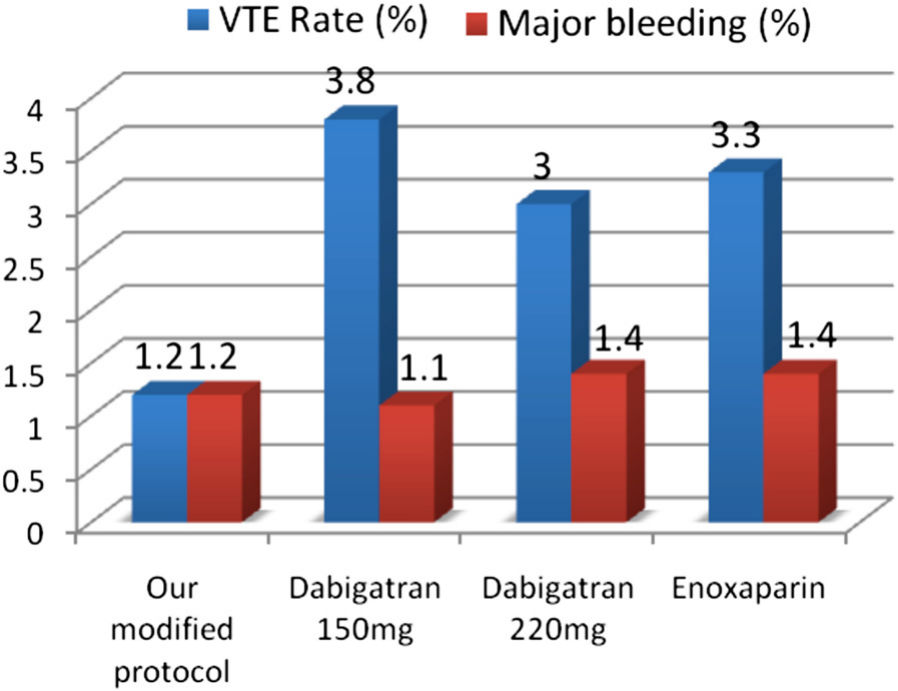
** Bar chart demonstrating results with our protocol versus those for pooled analyses from the major VTE trials **[15].

We had a DVT rate overall of 1.19% (4 patients) Figure 1. There were no PE’s. The majority of DVT’s occurred in patients who had undergone THR’s, with a rate of 1.96% (3 patients), and 1 patient in the TKR group with a rate of 0.54%. Table 1 shows that a large proportion of the DVT’s occurred in the cemented THR group (2/57 cemented THR versus 1/96 uncemented THR), this was not statistically significant (p = 0.67), chi squared test (SPSS). The DVTs occurred on an average of 34.5 days post operatively with a range of 21 – 45 days.

**Table 1:** Table showing summary results

	**Number of patients**	**Number of DVT/PE**	**% Patients DVT/PE**	Average Hb drop*	**% transfusion >1unit**	**% patients taken back to theatre**	**% Major bleed**	**% Clinically relevant non-major bleed**
TKR Uncemented	41	0	0	2.07	7.3	0	0	0
TKR Cemented	143	1	0.7	2.14	7.7	2.80 (n = 4)	2.80 (n = 4)	0.7 (n = 1)
THR Uncemented	96	1	1.04	2.82	13.5	0	0	1.04 (n = 1)
THR Cemented	57	2	3.51	2.81	21.1	0	1.75 (n = 1)	1.75 (n = 1)

* *Hb *= Haemoglobin.

The average haemoglobin drop for all patients was 2.46, as measured the morning following surgery. Haemorrhagic events were classified into two categories: major bleeds or clinically relevant non-major bleeds. Major haemorrhage constituted events that led to death, bleeding into critical organs, bleeding requiring a transfusion of more than 2 units of blood or bleeding that required a reoperation. Clinically relevant non major bleeds consisted of minor haemorrhage from body orifices (epistaxis, per rectal bleeding, macroscopic haematuria, wound haematomas that did not require reoperation) [12].

By these definitions there were 5 patients who suffered a major haemorrhage. There were 4 in the cemented TKR group that had to return to theatre for a haemarthrosis. The one patient in the cemented THR group who suffered a major haemorrhage required a transfusion of more than 7 units of blood products *intraoperatively* and was not therefore a consequence of VTE chemoprophylaxis.

The transfusion rates are set out in Table 1. With respect to the TKR’s who had a reperfusion drain, the transfusion rate referred to is not autotransfusion from their drain, but allogenic blood.

No THRs were taken back to theatre for haematoma or wound discharge. However 4 TKRs required washout for haemarthrosis. It is worth noting that none of these 4 patients taken back to theatre were being treated on Dabigatran at the time of developing a haematoma. There were no cases of deep infection. There was one death in our study. This patient had a cemented THR and died 13 months post operatively from a myocardial infarction.

On statistical testing (chi squared, SPSS) there was no statistically significant difference between total hip or total knee replacement subgroups in terms of transfusion rate (p = 0.09), return to theatre rate (p = 0.97) or those suffering a clinically relevant major bleed (p = 0.57).

## Discussion

The subject of VTE and prophylaxis became a national issue in January of 2010 when NICE released a document with prophylaxis algorithms for hospital patients, including sections specifically relating to lower limb arthroplasty [2]. Rightly or wrongly numerous orthopaedic departments found themselves having to alter their prophylactic regimes on the basis of this document, particularly to comply with the recommendations for anticoagulation to continue for 10-14 days post knee, and 28-35 days post hip arthroplasty.

Previously numerous anticoagulant regimes ceased on discharge of the patient, around day 5 post operatively [5,13,14]. One of the NICE recommended anticoagulants was Dabigatran Etexilate (Boehringer Ingelheim), which is an oral direct thrombin inhibitor. It had been the subject of large European multicentre trials (RENOVATE, REMODEL, REMOBILIZE) and had been demonstrated to be equivalent to subcutaneous Enoxaparin both in terms of clinical effectiveness and safety [7-9,15].

Three major prospective, randomized, double-blind non-inferiority trials have compared the efficacy and safety of Dabigatran (150 mg and 220 mg once-daily) starting postoperatively, with subcutaneous Enoxaparin, in patients undergoing hip (RE-NOVATE [6]) or knee arthroplasty (RE-MOBILIZE [9] and RE-MODEL [8]). In the RE-MODEL and RE-NOVATE trials, both doses of Dabigatran were as effective as Enoxaparin 40 mg once daily in reducing the risk of total VTE and all-cause mortality after hip [6] or knee arthroplasty [8]. Pooled analysis of major bleeding and the clinically relevant non-major bleeding indicated a similar bleeding profile with each Dabigatran dose, which was comparable with Enoxaparin [15]. The overall bleeding risk in patients older than 75 years and in patients with moderate renal impairment (Creatinine clearance >30 to <50 ml/min) was lower in the 150 mg subgroups compared with either 220 mg Dabigatran or Enoxaparin.

Friedman et al conclude that the pooled analysis of the three major prospective trials revealed no clinically relevant differences between Dabigatran at the 150 mg or 220 mg dosage regimes and Enoxaparin for the prevention of major VTE and VTE related mortality or for the safety profile [15] Figure 1.

In arthroplasty surgery there has always been a trade-off between the VTE prophylactic effects of anticoagulants versus reported increase in wound discharge, with the added concern that wound discharge potentially increases the risk of deep infection [16], which represents an unmitigated disaster in terms of outcome of the arthroplasty [17].

At the time of inception of our new protocol there were anecdotal reports from colleagues of increased problems of wound discharge post arthroplasty when Dabigatran was used exclusively. It was for this reason (supported by data from dose selection studies showing increased risk of bleeding on the day of surgery associated with Dabigatran [10]) that we decided to continue with our standard subcutaneous anticoagulant (Dalteparin) in the immediate post operative period, and not to prescribe Dabigatran until discharge, by which time the surgical wound had dried up (our patients are not discharged home until the dressing has been dry for 24 hours as a matter of departmental policy). It should be noted that our policies do not significantly alter lengths of stay of the patients. The results from this study illustrate our length of stay for patients post total hip and total knee replacement is 4.6 and 4.7 days respectively. This compares favourably with other studies in terms of short lengths of stay [18].

The benefits of an oral anticoagulant on discharge are self evident when compared to self administered subcutaneous injections, given the choice one would always choose the oral medication, all other factors remaining equal. Patient compliance is greater [4,5], and one can postulate the VTE prophylactic effect for ones patient population will be greater if they are actually taking the medicine.

Furthermore, Dabigatran has the advantage over low molecular weight heparins in that thus far there are no reported cases of significant allergic reactions to taking the medication, nor is there the risk of developing heparin induced thrombocytopenia, therefore negating the need for blood tests during the period of chemoprophylaxis.

Dabigatran comes with 2 dosage recommendations, the standard dose of 220 mg, and a lower dose for anyone over the age of 75 or with mild impairment of renal function (creatinine clearance 30-50 ml/min) [11]. This appeared to add a layer of complication for junior doctor prescription of discharge medication, with the scope for error. The decision was made therefore to prescribe the lower dose of 150 mg once daily to all patients for prophylaxis. A large proportion of our patients for arthroplasty are aged 75 years or over, and/or have a degree of mild renal impairment, so this seemed a sensible course of action.

This is a potentially controversial decision, because, despite the fact that the trials include a significant proportion of their patients being treated with the lower dose, they are all in the elderly or mildly renal impaired group, and the decision to treat all-comers on the lower dose could be considered in some ways to be an ‘off-licence’ use of the medication. The pooled analysis of the multicentre trials conclude that there are no clinically relevant differences between Dabigatran (220 mg or 150 mg) and Enoxaparin for the prevention of VTE or for the safety profile [15].

For the return to theatre for the washout of haematoma, two patients were on Dalteparin at the time. The other two cases were 23 and 64 days post operatively when they had finished treatment with Dabigatran. It should be noted that patients were on Dalteparin when the haemoglobin drop and transfusion rates were measured and Dabigatran had not yet been started. Our transfusion rates for TKR and THR are 7.6% and 16.3% respectively. The overall average transfusion rate is 11.6%. This is below quoted figures from other studies including Bell et al [19]. They report an overall 22% rate of transfusion in arthroplasty surgery when using Dalteparin for VTE prophylaxis. Other studies quote transfusion figures as high as 39%. This could be due to including patients undergoing revision surgery which are more likely to require blood transfusions.

There are some limitations to our study. It should be noted that it is assumed that patients who are prescribed the Dabigatran take the full course of medication and compliance has not actually been checked. It should be noted as mentioned earlier, one of the advantages of oral medication is improved compliance in taking the medication amongst patients. We do however feel that our protocol has promising results with valuable data and provides a growing body of evidence to the new oral anticoagulant and warrants further evaluation.

## Conclusion

Our treatment protocol is a novel practical approach for VTE prophylaxis in hip and knee replacement patients, combining subcutaneous Heparin in hospital with oral Dabigatran Etexilate after discharge. This approach shows promising data but no definitive evidence to warrant wide-spread use of this new regime. This data can act as a foundation for larger randomised clinical trials.

## Competing interests

The authors declare that they have no competing interests. There was no funding in this study.

## Authors’ contributions

PS was the lead author and investigator. SK and SS helped write the paper and helped with data collection. OJP was the senior author and developed the idea and helped write the paper. All authors contributed significantly to this paper. All authors read and approved the final manuscript.
